# Cooperative gene regulation by microRNA pairs and their identification using a computational workflow

**DOI:** 10.1093/nar/gku465

**Published:** 2014-05-28

**Authors:** Ulf Schmitz, Xin Lai, Felix Winter, Olaf Wolkenhauer, Julio Vera, Shailendra K. Gupta

**Affiliations:** 1 Department of Systems Biology and Bioinformatics, University of Rostock, Rostock, Germany; 2 Laboratory of Systems Tumor Immunology, Department of Dermatology, University Hospital Erlangen, Friedrich-Alexander-University Erlangen-Nuremberg, Germany; 3 Stellenbosch Institute for Advanced Study (STIAS), Wallenberg Research Centre at Stellenbosch University, Stellenbosch, South Africa; 4 Department of Bioinformatics, CSIR-Indian Institute of Toxicology Research, 226001 Lucknow, Uttar Pradesh, India

## Abstract

MicroRNAs (miRNAs) are an integral part of gene regulation at the post-transcriptional level. Recently, it has been shown that pairs of miRNAs can repress the translation of a target mRNA in a cooperative manner, which leads to an enhanced effectiveness and specificity in target repression. However, it remains unclear which miRNA pairs can synergize and which genes are target of cooperative miRNA regulation. In this paper, we present a computational workflow for the prediction and analysis of cooperating miRNAs and their mutual target genes, which we refer to as RNA triplexes. The workflow integrates methods of miRNA target prediction; triplex structure analysis; molecular dynamics simulations and mathematical modeling for a reliable prediction of functional RNA triplexes and target repression efficiency. In a case study we analyzed the human genome and identified several thousand targets of cooperative gene regulation. Our results suggest that miRNA cooperativity is a frequent mechanism for an enhanced target repression by pairs of miRNAs facilitating distinctive and fine-tuned target gene expression patterns. Human RNA triplexes predicted and characterized in this study are organized in a web resource at www.sbi.uni-rostock.de/triplexrna/.

## INTRODUCTION

MicroRNAs (miRNAs) are a well conserved and abundant class of ∼22 nt long functional RNA molecules that regulate the expression of most protein coding genes at the post-transcriptional level (1). Multiple predictions and growing experimental evidence suggest that many genes are targets of concerted miRNA regulation (1–3). Their expression is fine-tuned through a cellular context-dependent regulation by multiple miRNAs, where miRNAs can either induce translational repression or target mRNA degradation (4). Thereby, the miRNA-target regulation machinery can realize elaborate gene control functions, including noise buffering or homeostasis, and can ultimately mediate distinct target expression patterns appropriate to the demand of different biological processes (3,5,6). However, deregulated miRNAs have also been associated with the pathogenesis and the progression of many diseases, including cancer (7). Another remarkable aspect about miRNAs is that they provide a valuable source for diagnostic and prognostic markers for a growing number of human pathologies; especially those miRNAs found in body fluids (8,9). Besides, miRNAs became a popular subject for the design of novel therapeutic interventions. For details see the reviews by Seto (10) and Kasinski and Slack (11).

**Figure 1.**
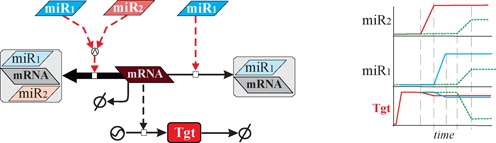
General principle of cooperative target regulations by pairs of miRNAs. The illustration on the left shows how a target mRNA can be repressed by either a single miRNA or by a pair of cooperating miRNAs. While in the first case miRNA and target form a duplex structure, the second case leads to the formation of a RNA triplex. On the right side, we illustrated the repressive effect on the target that is induced either by a single miRNA (red and blue lines) or by two cooperating miRNAs (green dashed line). Even if the expression of the cooperating miRNAs is only mildly up-regulated an enhanced repressive effect can be observed as compared to the cases where single miRNAs are highly up-regulated.

The phenomenon of cooperating miRNAs has, so far, not received extensive attention. It has been shown that pairs of miRNAs can synergistically regulate mutual targets to facilitate a more effective target repression (3,12–13), while the distance between the seed binding sites of a miRNA duet may affect the strength of target down-regulation (14). In this context, Sætrom *et al.* were able to determine an optimal seed site distance (13–35 nt) for miRNA cooperation (13). The concept of miRNA cooperativity implies a possible sophisticated mechanism of regulation of miRNA targets (Figure 1). For example, we have shown previously that higher quantities of miRNAs, as well as the phenomenon of synergistic target regulation, can work as an efficient noise buffer for target expression triggered by external stimuli (3). Furthermore, selective expression of cooperating miRNAs could be adopted by cells to facilitate distinctive and fine-tune gene expression patterns to meet the requirements in different biological scenarios (3,15). So far, only a few cases of synergistic target regulation by cooperating miRNAs have been identified and confirmed. Vella *et al.* were the first to validate this phenomenon in *Caenorhabditis elegans* (12). Later, this was confirmed in HeLa cells by Sætrom *et al.*, who also determined the seed distance constraint required for optimal target repression (13). In our own previous work we have analyzed this phenomenon and validated it in human SK-Mel-147 melanoma cells for the case of p21 which is regulated by miRNA-93 and miR-572 (3).

In the present work, we propose a workflow for the identification and analysis of novel animal RNA triplexes composed of two cooperating miRNAs and a mutual target mRNA. We have implemented the workflow and analyzed the human genome for cases of putatively cooperating miRNAs and their respective common targets. More specifically, we first made a whole human genome analysis in order to identify putative RNA triplexes based on predicted target sites and their respective seed site distance. Second, we analyzed the local secondary structure of the identified triplexes and computed the equilibrium probabilities of the inherent complexes (based on a partition function). Then, we determined their thermodynamic profiles by performing molecular dynamics simulations (MDS). Finally, we constructed a kinetic model of synergistic target regulation by cooperating miRNAs and simulated the target repression efficiency.

In summary, our results indicate that the phenomenon of cooperative miRNA-target repression is a prevalent mechanism of post-transcriptional gene regulation that affects thousands of human genes.

To make our results available to the public we have designed a database named as TriplexRNA. The database contains all predicted human target genes of synergistic miRNA regulation, including graphical illustrations of triplex secondary structures, their Gibbs-free energies (i.e. triplex-free energies) and predicted equilibrium concentrations. The database can be accessed at: www.sbi.uni-rostock.de/triplexrna/.

## MATERIALS AND METHODS

### Predicted and validated microRNA target interactions

miRanda (16) predicted miRNA binding sites in human target genes were extracted from the microRNA.org web site (hg19_predictions_S_C_aug2010.txt). We chose miRanda because of its high sensitivity and large relative overlap with other prediction algorithms (17,18). However, only those target sites of conserved miRNAs and with good prediction scores (mirSVR score) were considered. Experimentally validated miRNA-target interactions were derived from the miRTarBase database (19).

### Secondary structure and minimum free energy prediction

3′ untranslated region (UTR) sequences of target genes were extracted from the RefSeq gene track of the University of California Santa Cruz (UCSC) table browser (GRCh37/hg19; (20)). MiRNA sequences were extracted from the miRBase database (release 20; (21)). For structure prediction of the RNA complexes, sequences of mature miRNAs and the target subsequence that encloses both miRNA target sites were used. Secondary structure and triplex-free energy (TFE; a.k.a. Gibbs-free energy Δ*G*) of RNA triplexes were determined with the *mfe* tool from the NUPACK software package (22). In NUPACK the full partition function (except for pseudo-knots) of RNA complexes is computed in dilute solution. RNA triplexes as depicted in Supplementary Figure S1 were visualized using the command line version of the RNA structure drawing tool VARNA (version 3.9; (23)).

### Statistical analysis

All statistical analyses were performed using the R statistical computing software (version 3.0.1).

### 3D structure modeling and MDS

#### 3D model design

For the detailed 3D model of two miRNA-Argonaute hybrids (miR-20a/hAgo2) attached to one stretch of target 3′ UTR (NEURL1B mRNA) we retrieved the crystal structure of miR-20a/hAgo2 determined by Elkayam *et al.* (24) through X-ray crystallography from the Protein Data Bank (PDB id: 4F3T). All nucleotides and amino acid residues missing in the complex were modeled using Accelrys® discovery studio package. The accuracy of the modeled miR-20a was ascertained by calculating the backbone root-mean-square deviation of its selected bases from the template which was less than 4 Å. For the construction of initial complexes of mRNA with two miR-20a/hAgo2 hybrids, we used the PatchDock web server that performs docking between macromolecules based on shape complementarity principles (25) by providing mRNA-miRNA binding site information. From various docking poses suggested by the PatchDock server, we manually inspected every pose and selected the best complex which has maximum number of interacting bases between mRNA and miRNA along with a preserved seed binding. We optimized the structure using the smart minimizer algorithm in Accelrys® Discovery Studio. However, because of the unavailability of crystal structures of other miRNAs in complex with Argonaute (AGO) and computational feasibility we modeled the 3D structure of other RNA triplexes and their intrinsic RNA duplexes without considering AGO in the following way:

Initial tertiary structure of the RNA triplexes as well as their two intrinsic RNA duplexes (mRNA+miRNA_1_ and mRNA+miRNA_2_) were computed using the RNAComposer web server (26). RNAcomposer is a fully automated tool for constructing large RNA 3D structures from user provided secondary structure information. Therefore, the local secondary structures of the RNA complexes (bracket notation of concatenated RNAs units) were used as input. The retrieved 3D structures were edited using the *Build and Edit Nucleic Acid* tool in Accelrys^®^ Discovery Studio 3.5 to separate the three RNA units in the complex: First, the phosphodiester bonds were deleted that connect (i) the last base of mRNA and first base of miRNA_1_; (ii) last base of miRNA_1_ and first base of miRNA_2_. Second, the CapNucleotide function was used to change the phosphate group at 5’ of all the RNA units to a hydroxyl group so that two consecutive nucleotides of various RNA units cannot form phosphodiester bonds with each other in the complex.

After structure editing, the charmm27 force field was assigned to the RNA complexes, which is a superset of the charmm22 force field with the additional coverage for nucleic acids (27,28,29). The nucleic acids parameters of the charmm27 force field have been successfully used in many MDS studies involving RNA in the past (30–32).

The geometry of the initial 3D structure of RNA complexes was optimized using the Smart Minimizer protocol available in Accelrys^®^ Discovery Studio 3.5 to remove steric overlap that produces bad contacts. The Smart Minimizer was run for a maximum of 5000 steps with the Minimization RMS Gradient tolerance of 0.1 kcal/(mol x Å) to exit from the minimization routine in case the average gradient is less than or equal to the tolerance.

#### MDS setup

MDS were performed using the Simulation protocol available with Accelrys^®^ Discovery Studio 3.5. The optimized structure of the complexes was heated gradually from 50 to 300 K by scaling the velocity of each atom in a total of 10 000 steps with the iteration time step of 1 fs in order to prepare the system for the production run. After the heating phase, equilibration was performed to stabilize the system around the target temperature of 300 K by periodically reassigning velocities to each atom. The initial velocity of all the atoms was taken from the Maxwellian distribution at temperature 300 K by employing LeapFrog Verlet algorithms with the time step of 1 fs for 10 000 steps. RNA complexes were verified for their stability after the equilibration phase. For stable complexes, MD production simulations were performed for initially 100 ps duration. The canonical thermodynamic ensemble, i.e. constant-temperature and constant-volume ensemble (NVT) was selected for the production run. The motions of the molecules were recorded in the form of an output trajectory after every 1000 steps. Hydrogen bond monitors were applied to trajectory frames to investigate the stability of mRNA and miRNAs interactions during the course of simulation. The production phase for stable complexes after 100 ps of initial run was further extended for another 400 ps to check the stability of the RNA complexes.

All the simulation steps were carried out in the Generalized Born with a simple SWitching (GBSW) implicit solvent model for the better approximation of the solvent effect on the complex. GBSW has been made computationally less expensive by replacing its computationally expensive molecular surface approximation with a van der Waals-based surface with a smooth dielectric boundary (33). The implicit solvent dielectric constant was set to 80. Lower and higher cutoff distance for non-bonded interactions were set to 10 and 12 Å. The non-bonded lists were maintained for the atom pairs within the distance of 14 Å beyond which the non-bonded interactions were ignored. Input atomic radii were taken from Nina *et al.* as recommended for the charmm27 force field (34). SHAKE constraint was applied to fix all bonds involving hydrogen bonds.

### Complex equilibrium concentrations

The equilibrium concentrations in dilute solution for all considered complexes were computed using the tools *complexes* and *concentrations* (NUPACK package; (22)) and were based on an initial concentration of 100 nM (nanomolar) for each RNA species involved. The equilibrium concentrations of RNA triples were used for parameterizing the kinetic model of synergistic target regulation by pairs of miRNAs.

### Kinetic model calibration and simulation

Equilibrium concentrations of RNA triples and the potential energy values of inherent duplexes and the triplexes were used to characterize the association and disassociation rate constants of duplexes and triplexes, respectively.

The calibrated model was used to simulate the repression of the target genes by the cooperative miRNA pairs. To this end, the parameters accounting for the expression of the two miRNAs (*TF_miRNAi_*) were modulated in an interval of [10^−1^ 10^2^] which represents the down- and up-regulation of the miRNAs. The steady states of the target protein were computed for different combinations of miRNA expression. Due to the normalization mentioned above, the target expression levels computed were constrained between 0 (target is silenced) and 1 (no repression). Simulations were performed using COPASI version 4.11 (35).

## RESULTS

### Workflow for the identification and analysis of RNA triplexes

**Figure 2.**
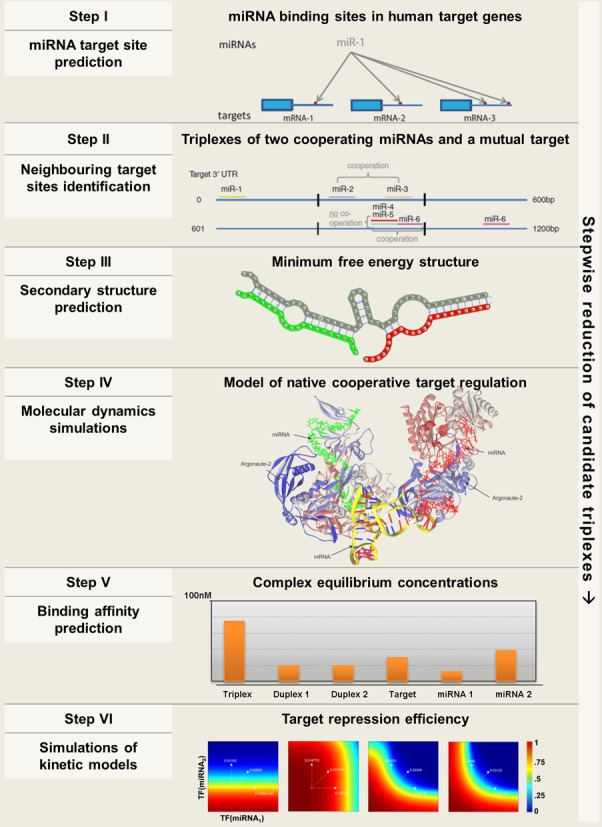
Workflow diagram. The diagram shows the six steps that we propose for the identification of gene targets that are efficiently regulated by two cooperating miRNAs. In each step the diagram indicates the approach used and the expected results. By advancing toward the end of the workflow only the most significant candidates from the initial set will remain.

Our proposed workflow for the identification and analysis of RNA triplexes in animal genomes includes six steps that sequentially increase the level of detail and confidence in the functionality of the predicted synergistic target regulation (Figure 2). These six steps include: (I) the identification of miRNA target sites in the 3′ UTR of gene targets; (II) the identification of putatively cooperating miRNA pairs with target sites in close proximity; (III) the prediction and analysis of the local secondary structure of the predicted RNA triplexes; (IV) the prediction of the 3D triplex structure including AGO and the determination of the triplex thermodynamic profile by MDS; (V) the determination of equilibrium concentrations (respectively binding affinities) of the inherent RNA complexes (monomers, duplexes and triplexes) and (VI) the determination of the target repression efficiency by simulations of a kinetic model.

In the following paragraphs we motivate and discuss each of the six steps in the computational identification and analysis of RNA triplexes. We then show how to implement the workflow and apply it on the human genome for the identification of cooperating human miRNAs and their mutual target genes.

#### Step 1 – miRNA target site identification

In this step target sites of miRNAs in mRNA transcripts are predicted. Functional target sites are typically found in the 3′ UTR of the mRNA. There is a plethora of miRNA target prediction algorithms available. Some use sequence, contextual, structural and/or evolutionary constraints for their predictions. Others are trained with experimental data from miRNA transfection/knockout experiments. Different miRNA target prediction approaches have been reviewed, for example, in (34,36–37).

Obviously, the most reliable target sites can only be inferred from experimental evidence. Approaches for the experimental validation of miRNA target sites are discussed in (38).

#### Step 2 – Target site distance determination

Target sites with seed site distances ranging between 13 and 35 nt in the 3′ UTR of target mRNAs are identified (13). These neighboring target sites are considered the basis for synergistic target regulation. Therefore, miRNA pairs that can hybridize with these sites can induce an enhanced target repression by a cooperative action on their mutual target. Computational predictions suggest that many genes have multiple, sometimes dozens of miRNA target sites in their 3′ UTR. Based on that, one can also detect a large number target sites in close proximity. However, it is likely that not all of the so detected putative RNA triplexes will be functional. Rather, one will retrieve a significant number of false positive predictions. It is, therefore, necessary to perform a more comprehensive analysis of target site pairs and their respective miRNAs in order to identify functional RNA triplexes in terms of synergistic target regulation and to minimize false positive predictions. The following steps in our workflow provide an *in silico* analysis pipeline which can be used to enhance accuracy in the search for candidates of synergistic target regulation and reduce the risk of experimental failure in the attempt to validate functional triplexes.

#### Step 3 – Secondary structure prediction and analysis

In the third step, the local secondary structure of putative RNA triplexes is predicted. One reason to do this is the possibility to see if the seed bindings of both miRNAs are being preserved in the minimum free energy structure of the triplexes (Supplementary Figure S1). The seed binding has been described as a crucial factor in functional miRNA-target interactions (39,40). Furthermore, from the predicted free energy one can evaluate the thermodynamic stability of an RNA complex. Hybridization energy values are frequently used as feature in algorithms for the identification of functional miRNA-mRNA duplexes (41,42). Thus, more stable triplexes may likewise enable more efficient target repression.

Due to the importance of structure stability for triplex function, we further propose a more comprehensive approach to predict the energy profiles of RNA triplexes, which builds on the secondary structure.

#### Step 4 – Molecular dynamics simulation

The fourth step of our workflow proposes the prediction and analysis of detailed 3D models of cooperative target regulation composed of two miRNA-AGO hybrids attached to the 3′ UTR of the target mRNA, followed by thermodynamic profiling by performing MDS. For a realistic model we require the crystal structure of the miRNAs in complex with AGO. As the crystal structure is unavailable for most miRNA-AGO complexes we suggest modeling RNA triplex structures without incorporating AGO instead. Thereby, the computational complexity is reduced and analytical throughput will be increased. In this case, the tertiary triplex structure is predicted based on the local secondary structure. Methods for template-based and template-free RNA 3D structure modeling have been reviewed in (43). Based on the MDS those RNA triplexes can be identified that are thermodynamically more stable than their inherent mRNA-miRNA duplex structures.

Results from this and the following step are pre-requisites for the prediction of the target repression efficiency and the synergistic effect achieved by the cooperating miRNAs.

#### Step 5 – Equilibrium concentration prediction

Here, we suggest predicting the equilibrium concentrations (binding affinities) of the inherent monomers and complexes (duplex and triplex). A partition function algorithm can be used, in case of a fixed volume (of dilute solution), to compute the equilibrium probability distribution of each possible complex that can be formed by the miRNA pair and its target (44). Thus, equilibrium concentrations of single molecules, duplexes and triplexes are computed based on a given initial concentration. The results are used to infer, whether molecular binding affinities will predominantly lead to the formation of RNA triplexes which may be required for cooperative miRNA target regulation.

#### Step 6 – Repression efficiency calculation

Ultimately, we suggest estimating the target repression efficacy and the synergistic effect achieved by the cooperating miRNAs by performing mechanistic model simulations. Here, a mechanistic model of the involved reactions, including RNAs syntheses, complex formations/dissociations and degradations, is used to simulate target steady states for different concentrations of the regulatory miRNAs. By comparing different scenarios (as described in the case study further below) one can determine if a miRNA pair cooperates in the target regulation (synergistic phenomenon) or, in contrary, just coregulates its mutual target (additive regulation).

We have implemented and applied the proposed workflow in order to identify all human RNA triplexes composed of two cooperating miRNAs and a mutual target. This case study will be described in detail in the following paragraphs.

### Identification and analysis of human RNA triplexes

We derived a collection of putatively cooperating miRNAs and their mutual targets by first extracting predicted miRNA target interactions from the microrna.org repository, which is based on the miRanda target prediction algorithm (16,45). The miRanda algorithm, which uses sequence composition, conservation and thermodynamic stability as criteria for predicting miRNA target sites, is a rather sensitive method, but has the highest relative overlap with predictions from other algorithms (36). Therefore, we consider miRanda as a representative of the majority of existing miRNA target prediction algorithms.

#### Seed site distance

**Figure 3.**
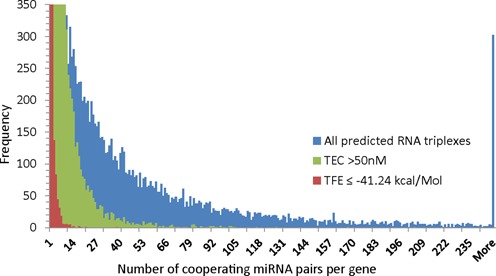
Histogram of triplex counts per single gene target in the human genome. Based on predicted miRNA-target interactions and the constraint for putative triplexes to be formed when seed site distances are in the range of 13–35 nt we can observe that most genes can form dozens of triplexes with pairs of cooperating miRNAs, while in some cases even more than hundred triplexes are possible with a single gene (blue bars; *y*-axis has been truncated; frequencies for values >250 have been added up at the right-most tick mark on the *x*-axis). However, this is not a realistic scenario and therefore we suggest to apply cut-offs for the predicted triplex equilibrium concentrations (e.g. TEC > 50 nM, green bars) and for the predicted TFE (e.g. TFE ≤ −41.24 kcal/mol, red bars). This leads to a reduction in the number of predicted triplexes as per gene and results a set of stable high-confidence triplexes composed of two cooperating miRNAs and a mutual target.

Sætrom *et al.* disclosed a distance constraint of 13–35 nt for target sites of cooperating miRNAs (13). Based on this constraint and the predicted miRNA target interactions we identified 17 259 human genes (out of 19 898; ∼87%) as putative targets of cooperative post-transcriptional regulation by 29 060 distinct miRNA pairs. See the frequency of triplex numbers predicted for genes in Figure 3. In the course of the proposed workflow the number of target genes decreased with each of the following steps, toward a smaller subset of high-confidence targets.

We plotted histograms of the seed site distance frequencies for different triplex structure conformations in Supplementary Figure S2. However, among the identified target site pairs we did not observe any preferential seed distance, despite a frequency decline toward larger distances.

#### Conservation

There is evidence that targets with conserved seed sites face stronger miRNA-mediated repression (1), which is in line with the observations made by (46) who found an enrichment of down-regulated targets with conserved miRNA binding sites in miRNA transfection experiments. This suggests that target site conservation is a valid determinant for functional miRNA-target regulation. Surprisingly, we observed that triplexes with pairs of strongly conserved target sites tend to have lower predicted triplex equilibrium concentrations, i.e. in these scenarios triplex formation is not favored (Supplementary Figure S3; conservation based on PhastCons score; (47)). In contrary, weakly conserved target site pairs tend to have higher predicted triplex concentrations. One possible explanation for this observation is that miRNAs with strongly conserved target sites are inherently effective regulators of the corresponding target gene, whereas weakly conserved target sites are often non-functional and require support by a second proximate miRNA target site to enhance, through synergy, the repression of the target. Interestingly, this trend cannot be observed for the mRNA-miRNA duplexes that may arise from RNA triples (Supplementary Figure S3).

However, by solely considering the predicted miRNA target sites and the seed distance constraint we found that each miRNA is putatively cooperating with almost all (or all) other miRNAs in the regulation of some target mRNA. Obviously, these criteria are not enough for the identification of functional RNA triplexes. Rather, we have to determine if RNA triples form thermodynamically stable triplexes and if there exists a strong binding affinity among the involved molecules. These criteria are additional pre-conditions for a cooperative target regulation. Therefore, we determined the local secondary structure for the predicted triplexes and analyzed it for conformational and architectural patterns.

#### Segregating non-functional triplexes by free energy and free energy gain values

Secondary structure predictions revealed RNA triples which are energetically in favor of a miRNA-mRNA duplex and leave one miRNA isolated. We discarded these triplexes from further analysis (see the Supplementary Materials for more information on structural conformations of RNA triplex secondary structures). This reduced the number of putative target genes from 17 259 to 15 062 genes. Along with the local secondary structure we predicted the TFE, respectively, Gibbs-free energy (Δ*G*_triplex_). According to Muckstein *et al.* the efficiency of RNA interference correlates with the binding energies of siRNAs/miRNAs to their respective mRNA target (48). This suggests that thermodynamic stability of RNA triplexes may be a crucial determinant for miRNA cooperativity and the strength of synergistic target repression. Thus, the number of candidates for synergistic target regulation will certainly reduce by applying a filter that discards RNA triplexes with a predicted TFE higher than a certain cut-off. For example, the average number of cooperation partners per miRNA drops from 242.29 (no cut-off) to 23.0 by applying a cut-off at Δ*G*_triplex_ = −41.24 kcal/mol, while the number of targets is reduced to 1779 genes only. This cut-off value is 3 standard deviations below the mean TFE of all predicted RNA triplexes (*Z* = 3). This *Z*-value was proposed in (49) for miRNA-target duplexes.

Furthermore, we compared the TFE values of the predicted triplexes with the conceivable duplexes formed by the components of a RNA triple (ΔΔ*G* = Δ*G*_triplex_ − Δ*G*_duplex_min_). In 674 310 predicted RNA triplexes, we found only one case (miR-137::MYB::miR-374a) where duplex formation was slightly beneficial in terms of free energy (Δ*G*_triplex_ = −9.56; ΔΔ*G* = 0.22). However, in this case seed bindings of the involved miRNAs were not preserved in the predicted triplex structure. Nevertheless, we observed that the free energy gained through triplex formation can be small, e.g. in case of miR-1::PROS1::miR-320b (Δ*G*_triplex_ = −24.16; ΔΔ*G* = −0.48) but also rather high as in case of miR-197::CNTN5::miR-320a (Δ*G*_triplex_ = −43.06 kcal/mol; ΔΔ*G* = −24.08 kcal/mol). Thus, determining the TFE gain (ΔΔ*G*) has been used as another step toward the identification of stable and effective RNA triplexes.

The RNA triplex structure that includes miRNA-miRNA hybridization is typically built at the cost of seed site preservation, i.e. for at least one of the two miRNAs the 5′ end is not hybridizing with the designated seed site in the target but with the 3′ end of the other miRNA (see, for example, Supplementary Figure S4D). As seed binding is crucial for efficient target repression (39,40), we discarded this structural conformation from the set of RNA triplex candidates, which reduces the number of target genes to 14 956, when no other filter is applied. By discarding all other triplexes that have no preserved seed bindings the number of targets was reduced to 14 527.

#### The role of AGO proteins in RNA triplexes

MiRNAs are embedded in the RNA-induced silencing complex when exerting their function. More specifically they are bound to AGO proteins which they guide to their designated target sites. To know more about the interplay of two cooperating miRNAs with AGO and their mutual target mRNA we adopted 3D modeling and investigated the impact of AGO on the RNA triplex’ binding affinity. Therefore, we utilized the structure of human argonaute-2 (hAgo2) in complex with miR-20a previously generated by Elkayam *et al.* (24) at 2.2 Å resolution (PDB ID: 4F3T) as basis for 3D modeling. Among the previously identified putative targets of synergistic miRNA regulation, we found two mRNAs, namely, NEURL1B and ZBTB24, with two miR-20a binding sites that reside in close proximity. We selected the NEURL1B-miR-20a-miR-20a triplex to observe if AGO proteins have any impact on the stability of miRNA-mRNA hybridization in neighboring binding sites.

**Figure 4.**
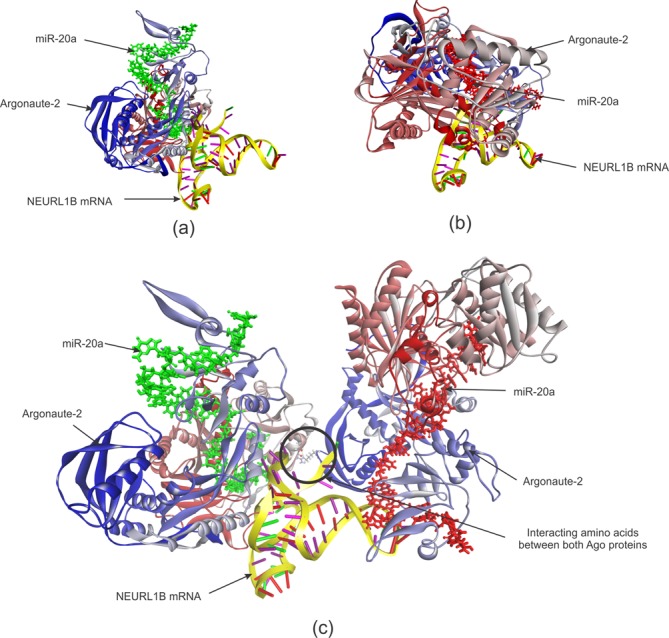
NEURL1B mRNA and miR-20a/hAgo2 interaction. **(a)** miR-20a/hAgo2 complex hybridized to the first binding site in NEURL1B mRNA. **(b)** miR-20a/hAgo2 complex hybridized to the second binding site in NEURL1B mRNA. **(c)** One miR-20a/hAgo2 complex attached to each of the two neighboring binding sites in the NEURL1B mRNA. miR-20a as stick model is colored in green at the first binding site and colored in red at second binding site. Both AGO proteins are shown as solid ribbon and amino acid residues are colored in a continuous gradient from blue at the N-terminus through white to red at the C-terminus. NEURL1B mRNA 3′ UTR fragments are depicted as yellow backbone with ladder-shaped base pairs. In the RNA triplex shown in (c), a black circle indicates the position of the interacting amino acid residue (AGO1:THR555- AGO2:ARG110) between the two AGO proteins.

For the analysis we built three models: (i) miR-20a/hAgo2 attached to the first binding site in NEURL1B mRNA; (ii) miR-20a/hAgo2 attached to the second binding site and (iii) one miR-20a/hAgo2 complex attached to each of the two neighboring binding sites in the NEURL1B mRNA. All three 3D models are depicted in Figure 4.

Interestingly, we observed that the complex with two sets of miR-20a/hAgo2 bound to NEURL1B was more stable (potential energy: −61,822.58 kcal/mol) than both complexes with a single unit of miR-20a/hAgo2 (potential energy: −34,129.67 and −23,869.30 kcal/mol).

The 3D model of the RNA triplex in association with two AGO proteins suggests that it is the interaction between the two AGO proteins that provides additional stability for the RNA triplex (NEURL1B-miR-20a-miR-20a). As experiments by Sætrom *et al.* (13) have shown the optimal seed site distance for miRNA cooperativity is 13–35 nt; our results also indicate that if the seed sites are too close (<13 nt), there might be steric hindrance between the two AGO units, while in case the seed sites are too far apart (>35 nt), there may not be any interaction between two AGO proteins that would contribute toward the stability of the RNA triplex.

#### MDS to disclose unstable RNA triplexes

To further investigate triplex stability and thermodynamic profiles of selected triplexes we performed MDS. To this end, we selected candidates that represent extreme cases for the TFE value (3x low and 3x high Δ*G*) and the free energy gained by triplex formation compared to the inherent duplexes (3x low and 3x high ΔΔ*G*). The 12 selected candidates are listed in Table 1.

**Table 1.** RNA triplexes selected for 3D structure modeling and MDS.

Energy parameter	mRNA	miRNA1	miRNA2	Triplex energy (kcal/mol)	Free energy gain (kcal/mol)
high minimum free energy values	*RPS6KA5*	miR-410	miR-590–3p	-12.263	-4.782
	*HTRA2*	miR-374a	miR-374a	-11.363	-3.982
	*ZNF121*	miR-374a	miR-374a	-10.363	-4.682
low minimum free energy values	*EDA2R*	miR-125a-3p	miR-370	-57.963	-22.282
	*MUC1*	miR-145	miR-326	-55.963	-15.282
	*ABT1*	miR-214	miR-491–5p	-51.163	-16.182
high free energy gain	*GOLM1*	miR-296–3p	miR-330–5p	-47.263	-21.482
	*CCDC3*	miR-138	miR-551b	-55.263	-21.782
	*PLXNB1*	miR-197	miR-320d	-49.863	-23.482
low free energy gain	*NPHP1*	miR-194	miR-340	-16.963	-1.782
	*KAT2B*	miR-106b	miR-590–3p	-22.263	-1.682
	*C19orf69*	miR-190	miR-590–3p	-25.063	-1.582

Representatives from the three different secondary structure patterns have been selected based on the TFE value and the free energy gained through triplex formation. Structural patterns are discussed in detail in the Supplementary Materials.

Subsequently, we derived the duplex and triplex 3D structures for the selected candidates. As the crystal structure for miRNA-AGO complexes in these candidates is unavailable we modeled the RNA triplex structures without AGO. All the initial 3D models were optimized using the Smart Minimizer energy minimization protocol in Accelrys^®^ Discovery Studio to remove any of the steric overlaps that produce bad atomic contacts and to obtain the stable structure with minimum free energy. The energy minimized 3D models of RNA triplexes are provided as Supplementary PDB files 1–12.

We performed MDS experiments using the simulation protocol available with Accelrys^®^ Discovery Studio 3.5 to underpin the hypothesis that triplexes are thermodynamically more stable and thus favorable as compared to their inherent duplex structures. For this, we examined the hydrogen bonds formed between miRNA and mRNA strands and considered the complex stable as long as there is any hydrogen bond present during the production run of the MDS. We first calculated the time for which mRNA and miRNA were bonded in the duplex structures and then determined if the binding time increased in case of the triplex structure. Furthermore, we considered 100 ps as threshold time for RNA triplexes to be selected as potential cases of miRNA cooperativity. In summary, we defined the following constrains for candidates of functional RNA triplexes:

\begin{equation*} ST_{triplex} \ge 100ps;and \end{equation*}

\begin{equation*} ST_{triplex} > \min \left\{ {\begin{array}{*{20}c} {ST_{miR1\_duplex} } \\ {ST_{miR2\_duplex} } \\ \end{array}} \right. \end{equation*}

where $ST_{triplex}$ is the stability time, i.e. the duration for which both miRNA strands are attached to the mRNA target through hydrogen bonds; $ST_{miR1\_duplex} \;{\rm and}\;ST_{miR2\_duplex}$ denote the stability times of the miRNA_1_-mRNA and miRNA_2_-mRNA hybrids in the MDS production run. From our selected candidates these constraints were fulfilled by the three triplexes with the low minimum free energy values and the three triplexes with the strong free energy gain (Table 2).

**Table 2.** Stability time of RNA complexes during MDS production run (in pico seconds).

Gene	In RNA duplex		In RNA triplex	
	mRNA + miRNA_1_	mRNA + miRNA_2_	mRNA + miRNA_1_	mRNA + miRNA_2_
*RPS6KA5*	139	145	62	92
*HTRA2*	72	72	116	75
*ZNF121*	250	250	58	91
*EDA2R*	206	468	218	449
*MUC1*	249	192	179	500
*ABT1*	327	338	239	399
*GOLM1*	500	395	118	500
*CCDC3*	500	479	500	500
*PLXNB1*	333	74	207	387
*NPHP1*	113	203	47	44
*KAT2B*	106	323	500	100
*C19orf69*	257	214	74	196
*CDKN1A*	42	301	126	473

Complexes, where the triplex is stable for more than 100 ps and the stability time of any of the miRNAs exceeds that of their corresponding duplexes, were considered as potential triplex to show miRNA cooperativity (in shaded rows).

Therefore, the simulation results for most of our selected candidates met our expectations, i.e. that triplexes with low TFE value and strong free energy gain are more stable than their inherent duplexes. This again supports our hypothesis that TFE and free energy gain are crucial factors for determining whether or not a triplex formed by a target gene and a cooperative miRNA pairs is functional.

However, one candidate that was unexpected to fulfill these constrains was the triplex involving the gene *KAT2B* and the miRNAs miR-106b and miR-590–3p which has little free energy gain but a moderately low TFE value (−22.26 kcal/mol). The complex stability times of the selected RNA triplexes are shown in Table 2. For illustration purposes we captured the molecular dynamics for the case of one stable RNA triplex (miR-138::*CCDC3*::miR-551b) and one unstable RNA triplex (miR-374a::*HTRA2*::miR-374a; see Supplementary Videos S1 and S2).

To demonstrate a proof of principle we repeated the described protocol for the RNA triplex composed of miRNA-93::*CDKN1A*::miRNA-572 for which we previously validated the synergistic effect the two miRNAs show in the repression of their mutual target (13). Our simulations show that the duplexes miRNA-572::*CDKN1A* and miRNA-93::*CDKN1A* are stable for 42 and 301 ps in the production run, while in the RNA triplex the stability times of miRNA-572 and miRNA-93 increase to 126 and 473 ps, respectively. This observation clearly indicates that the triplex formation is favored over the duplex in this particular case and also that estimation of stability time can be used as one parameter to filter triplexes with the potential to show miRNA cooperativity.

The results are presented in short simulation videos in the Supplementary Videos S3–S5. In these three simulations we compared the thermodynamic profiles of three possible complexes (i) miR-93::*CDKN1A* duplex; (ii) miR-572::*CDKN1A* duplex and (iii) miR-93::*CDKN1A*::miR-572 triplex.

In summary, MDS can be used to further discriminate between thermodynamically stable and unstable RNA triplexes. However, these simulations require a significant amount of time (∼24 h for 100 ps MDS in an implicit solvation box on a workstation with 12 cores, 2.3 GHz and 16GB RAM) and, therefore, we did not apply them for entire genome analysis.

#### Triplex formation is dependent on equilibrium distributions

For each complex species (i.e. monomer, duplex and triplex) of a RNA triple, equilibrium concentrations were computed using a partition function algorithm following the approach described in (44). This enabled us to estimate the association rates (affinity of the involved molecules) for triplex formation. These were used later for the parameterization of a kinetic model of miRNA cooperativity (see next subsection).

By defining an initial concentration for the involved molecules (e.g. 100 nM each) the partition function algorithm computes equilibrium concentrations (0–100 nM) for all possible complex species (Supplementary Figure S5). We concluded that only those RNA triples with a high triplex formation probability, i.e. high triplex equilibrium concentration, are likely to exhibit cooperative miRNA-target regulation. More specifically, only in case the majority of RNA molecules (>50%) is hybridized in RNA triplexes composed of two miRNA and one mRNA species cooperative target regulation can be expected. Thus, by defining a threshold for the predicted triplex equilibrium concentration, favorable RNA triplex candidates can be identified. When we applied a threshold of >50 nM for the equilibrium concentration of triplexes composed of an mRNA target and two miRNA species, we reduced the number of putative human RNA triplexes by ∼85% from 674 310 to 98 073, when no other constraint was applied (82 481 with preserved seed binding), involving 11 654 (10 883) target genes.

#### Repression efficiency simulated by a kinetic model of miRNA cooperativity

For the last step in our workflow, we developed a kinetic model using ordinary differential equations (ODEs) to analyze the synergistic regulation by pairs of miRNAs and the consequences for target gene dynamics. The model is based on our previous work (3) and accounts for all processes and the involved molecules in miRNA-mediated gene repression, including the formation of duplexes by the target mRNA and one miRNA, the formation of a triplex by the target mRNA and two miRNAs and the process of disassociation of the duplexes and the triplex (Supplementary Figure S6). See Supplementary Materials for a detailed description of the model.

**Figure 5.**
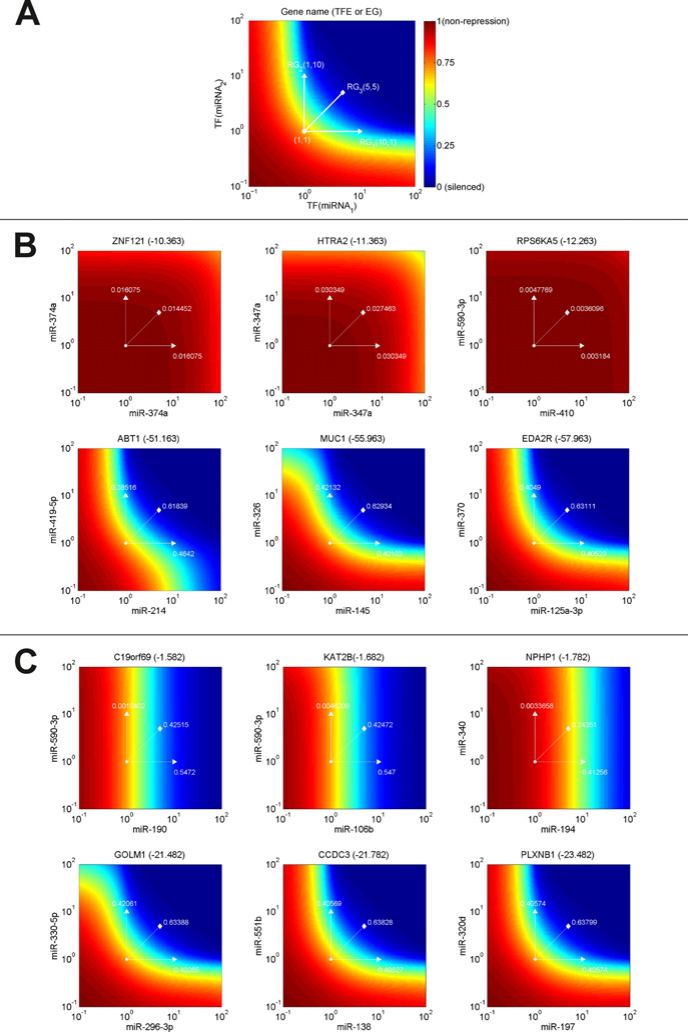
Simulation of gene repression by cooperative miRNA pairs. (A) Illustrative plot to explain simulation results. For combinations of miRNA expression rates in specific intervals [10^−1^ 10^2^] we computed the steady states (SS) of the target protein, which are color coded in a rainbow scale from red (full expression) to blue (fully silenced). Besides, we computed the RG of a target gene for three scenarios: (i) and (ii) the expression of either miRNA_1_ or miRNA_2_ is highly up-regulated (RG_1_ = SS_(10,1)_-SS_(1,1)_ or RG_2_ = SS_(1,10)_-SS_(1,1)_), and (iii) both miRNAs are being modestly up-regulated (RG_3_ = SS_(5,5)_-SS_(1,1)_). The higher the RG the stronger the repression effect on the target gene. The title in each plot contains the official gene symbol of the target and its TFE (Δ*G*) or energy gain (ΔΔ*G*). (B) Simulation results for triplexes with high (upper panel) and low (lower panel) TFE. (C) Simulation results for triplexes with low (upper panel) and high (lower panel) EG.

Next, we used the model to simulate target gene repression by cooperating miRNA pairs. We computed the steady states of the target genes by applying a range of different synthesis rate constants for the regulatory miRNA pair (see Materials and Methods section for details). Furthermore, to show the cooperative effect of the selected miRNA pairs we computed the repression gain (RG) of the target genes for three scenarios: (i) and (ii) strong up-regulation of one of the two miRNAs, and (iii) moderate up-regulation of both miRNAs in combination (see Materials and Methods section for details). The results for the same exemplary triplexes as for the MDS are presented as surface plots in Figure 5. The simulations show different patterns of gene repression by cooperative miRNA pairs for cases of high (upper panel; Figure 5B) and low TFE (lower panel; Figure 5B). For triplexes with high TFE we observed only weak repression ability, which is due to their thermodynamically unstable structure. In contrary, targets involved in stable complexes with cooperating miRNAs (lower panel) are efficiently repressed. We introduced values for the RG which represents a measure for the efficiency gained in target repression by either overexpressing a single miRNA (RG_1_ and RG_2_) or by synergistic miRNA-target regulation (RG_3_; see Figure 5 for details). In the upper panel of Figure 5B (representing high TFEs); RG_3_ is only insignificantly higher than RG_1_ and RG_2_. However, in the lower panel (representing low TFEs) RG_3_ is considerably higher than RG_1_ and RG_2_. We can conclude that a notable synergistic effect in target regulation by cooperating miRNA pairs can be observed in triplexes with thermodynamically stable structures (Figure 5B).

Similarly, the simulations showed different patterns of gene repression for cases of high and low free energy gain (ΔΔ*G*; Figure 5C). In the upper panel with triplexes having lower free energy gains compared to their inherent duplexes we found cases of independent target regulation with no synergy between the two miRNAs. In these cases the target can be repressed by only one of the two miRNAs. In the lower panel with RNA triplexes that have higher free energy gain compared to their inherent duplex structures, simulations show an efficient target repression through the individual miRNAs and a considerable synergistic effect by collective target regulation by both cooperating miRNAs ($RG_3 \gg RG_{1,2}$).

Taken together, Δ*G* and ΔΔ*G* of the triplexes formed by the mRNA and cooperative miRNA pairs are two important factors influencing the cooperative effect in miRNA-mediated target repression.

### A database of RNA triplexes

In our study, we performed a whole human genome analysis in order to identify putative RNA triplexes and characterize their structural and thermodynamic properties. To make our results available we designed a database of RNA triplexes formed by target genes and pairs of synergistically acting miRNAs. Besides, we referenced given experimental evidence supporting pairwise miRNA-target interactions, which we extracted from the miRTarBase database (36).

Through a web interface the RNAtriplex database can be queried for either miRNAs or genes to receive information about their involvement in RNA triplexes. Furthermore, users can sort the results; define a threshold for the TFE value or filter out triplexes with non-conserved seed binding. Moreover, comprehensive information about molecule sequences, genomic coordinates, triplex-free energies, experimental evidence, secondary structures and repression efficiency simulations can be retrieved and alternatively be extracted via the RESTful interface to the database, which is useful for the programmatic extraction of data.

## DISCUSSION

With the discovery and functional characterization of non-coding gene transcripts we now know that a plethora of different non-coding RNAs can mediate the regulation of gene expression (50). However, there is still a lot of work ahead to understand the transcriptomic complexity and all facets by which genes are being regulated (51). In this context the class of miRNAs crystallized in the last years as an omnipresent regulator of animal gene expression at the post-transcriptional level (52).

In the presented work, we studied the phenomenon of synergistic target regulation by pairs of cooperating miRNAs. This phenomenon can foster, at the post-transcriptional level, an effective and fine-tuned regulation of gene expression and can buffer efficiently noise coming from external stimuli (3).

### An integrative workflow for the identification of RNA triplexes

By integrating several computational approaches, ranging from RNA hybridization prediction, over structure modeling and MDS, to kinetic modeling, we designed a systematic and comprehensive analytical workflow for the identification of animal RNA triplexes composed of two cooperating miRNAs and their mutual target mRNA.

In the proposed workflow RNA triplexes are derived from miRNA target predictions followed by seed site distance filtering. Local secondary structure prediction and minimum free energy calculation are used to narrow down RNA triplexes that are likely to be functional. The analysis is further substantiated by MDS that are used to determine the energy profile of RNA triplexes. Furthermore, a mathematical model of synergistic target regulation is developed to infer target repression efficiencies mediated by cooperating miRNA pairs.

We implemented the workflow to predict human RNA triplexes composed of two cooperating miRNAs and their mutual targets. Results of our genome-wide analysis suggest that synergistic target regulation is a common phenomenon in human. We identified 15 062 human candidate genes that may be target of synergistic miRNA regulation. Some of the identified cooperating miRNA pairs have more than just one mutual target gene. For example, the miRNA couple composed of hsa-let-7g and hsa-miR-376c has most targets in common (14 mutual target genes: *AMMECR1L*, *CYP26A1*, *ZFYVE26*, *MUC7*, *FUS*, *TMEM38B*, *CWH43*, *CWH43*, *LIN28B*, *ZNF823*, *DVL3*, *SRGN*, *INPP5A*, *HDAC8*). However, in the course of the workflow the overall set of candidate triplexes can be reduced to a subset of high-confidence triplexes, likely to be functional. For example, the number of cooperation partners per miRNA reduced significantly when stringent filtering criteria were applied (Supplementary Figure S7).

We conclude that the outlined workflow is a comprehensive way for the *de novo* identification and characterization of RNA triplexes representing cooperative target regulation by two miRNAs.

### Prediction of human RNA triplexes—sensitivity versus specificity

In our analysis we used miRNA-target predictions from the miRanda algorithm (briefly described in the Supplementary Materials), a sensitive method that reflects a good cross-section of other existing methods, because it has the highest relative overlap with the predictions from other algorithms. Nevertheless, it has to be noted that miRNA-target interactions derived from miRanda may contain a significant number of false-positive predictions (36,53). We reduced these by considering only conserved miRNAs and target sites with good prediction scores for further analysis (mirSVR score: $ \le - 0.1;$ (16)). It has to be noted that miRNAs with binding sites in the 5′ UTR and the coding sequence of target mRNAs have been reported (17–18,54). However, experimental validation for synergistic target regulation by pairs of miRNAs has been carried out so far only for cases in which miRNA binding sides reside in the 3′ UTR of a mutual target. Although synergistic target regulation may also work in other regions than the 3′ UTR, we restricted our analysis to putative RNA triplexes in this region only.

In order to further reduce the search space for cooperating miRNAs and their respective targets, one can restrict the considered miRNA-target interactions to those that were computed by several algorithms using different prediction parameters and/or those being experimentally supported. We used the latter approach to generate a subset of RNA triplexes with partial experimental support based on validated miRNA-target interactions archived in the miRTarBase database (Supplementary Excel File; (19)). The total number of triplexes with experimental support is 952, involving 252 human genes. In this subset, 346 miRNA binding sites were supported by the recently developed experimental technique for the high-throughput identification of exact miRNA binding sites CLASH (crosslinking, ligation and sequencing of hybrids) (54,55).

On the other hand, stringent filtering criteria may result in a reduced sensitivity, i.e. some RNA triplexes may not be detected (false-negative predictions). It is, however, possible to expand the range of predicted miRNA target sites by considering also non-conserved miRNAs, respectively, target sites or lower prediction scores as well as the union of predictions from other algorithms. But this will certainly also increase the number of false-positives.

Nevertheless, for our case study in the human genome we compromised between sensitivity and specificity, and chose a miRNA target prediction algorithm that represents a good cross-section of predictions from most of the other existing algorithms.

### Seed binding determines efficacy of cooperative regulation

Previous studies have shown that the distance of seed sites can be used to discriminate non-cooperative from cooperative miRNAs that post-transcriptionally repress target genes in a synergistic manner (12,13). We adopted the results from the analysis performed by Sætrom *et al.* who found that maximal repression of a reporter gene construct by the miRNA let-7 was achieved when seed regions of a pair of let-7 target sites were 13–35 nt apart (13). However, within this range we could not identify any distance that is superior compared to the others in terms of TFE, triplex equilibrium or RG (data not shown).

Moreover, our predictions indicate that in many cases the seed binding of one or both miRNAs is not preserved upon complex formation, i.e. in the local secondary structure of RNA triplexes. In these cases MDS demonstrated that these triplexes are rather unstable (data not shown).

Of note, in this work we neglected the aspect of site accessibility which is considered in some target prediction algorithms, where the secondary structure of the full length 3′ UTR (56,57) or even the entire mRNA (28) was predicted for the estimation of site accessibility and calculation of the energetically favorable arrangement. We were, however, more interested in a good structure prediction accuracy which inevitably decreases with sequence length (58).

### RNA triplex structures—from 2D to 3D

We adopted the notion that RNA complexes are more stable when they have a comparably low binding free energy. This criterion is used in established non-coding RNA and miRNA-target prediction algorithms to discriminate putatively functional from non-functional structures. For local secondary structure prediction and minimum free energy calculation we used the tools *mfe* and *complexes* from the NUPACK package, a software suite developed for the analysis and design of nucleic acid complexes (22). It has to be noted that, for computational feasibility, we excluded pseudo-knots from our structural predictions which is described as a NP-hard problem in (59). The secondary structure predictions were used as a means for the high-throughput (whole genome) identification of RNA triples that are in general able to form stable RNA triplexes. We want to emphasize that secondary structure prediction is an essential part in the methodology adopted by the majority of miRNA-target prediction algorithms, and hence in our approach. In this context, the base pairing between mRNA and miRNAs in the secondary structure and the computed thermodynamic stability is crucial. Furthermore, due to 2D structure predictions we get instantaneous insight into seed binding preservation after folding and the overall site accessibility.

A better reflection of the native miRNA cooperativity process is certainly achieved by constructing detailed 3D models of cooperative target regulation that include two miRNA-AGO hybrids attached to a target 3′ UTR. However, a reliable calculation would require the data derived from the crystal structures of the miRNAs in complex with AGO. So far only one such structure is known (24). Furthermore, such a model increases the computational complexity drastically. In this paper, the methodology proposed is a compromise between the level of detail and the computation effort required to generate and simulate 3D models of miRNA cooperativity. Nevertheless, we foresee that the combination of better computational capabilities, the availability of more data from crystal structures of the miRNAs in complex with AGO and the development of customized protocols for the *ab initio* construction of 3D models will make possible in the next future the construction of detailed 3D models of cooperative target regulation that include two miRNA-AGO hybrids.

Due to computational complexity and unavailability of miRNA-AGO complexes, we decided to design 3D models of RNA triplexes without considering AGO. To our knowledge, there is no tool or protocol available for the *ab initio* construction of 3D models of mRNA–miRNA triplexes. Our 3D models are based on the 2D structures predicted by *mfe*. More specifically, 2D triplex structures give raise to possible interaction sites which are used for building the 3D model. Frequently cited RNA 3D structure modeling tools (such as RNAcomposer, MC-SYM, etc.) use single stranded RNA sequences and secondary structure folding information in dot bracket notation for their predictions. MC-SYM, for example, to model the RNA duplexes, artificially introduces a GAAA tetraloop between two strands of RNA and thus models the 3D structure of large single stranded RNA. Thereafter, the tetraloop is removed again to separate the two strands. We also modeled mRNA-miRNA triplexes as a single strand and then separated mRNA and two miRNAs strands by manually deleting the bonds. This approach is the same as in (60) with the exception that we did not introduce any artifact by including an extra nucleic acid sequence. The strategy was adopted to ensure a consensus between 3D structures generated by RNAcomposer and the secondary structure pattern predicted in the previous step of our workflow. Structure editing steps in our presented workflow are necessary to separate the three RNA strands. Likewise, the removal of GAAA tetraloops in MC-SYM entails these editing steps. To further optimize the geometry of the RNA triplexes, we used the energy optimization protocol available in Accelrys® Discovery Studio 3.5 to remove any steric overlap that produces bad contacts.

In summary, we generated a more realistic model of mRNA-miRNA interaction by only considering hydrogen bonds between the two RNA species and not by introducing an artificial intermediate nucleotide sequence for structural analysis and MDS studies. The same strategy was reported in other studies (61,62). The workflow to construct the tertiary structures of mRNA-miRNAs triplexes is summarized in Supplementary Figure S8.

3D structures of RNA triplexes form the basis for MDS. MDS are an important technique to generate a model of a structure's motion and to perform the time-continuous analyses of various structural and energetic properties of molecule complexes due to small- and large-scale atomic movements. Moreover, they can be used to observe the solvent effect on the structure, energetics and dynamics of biomolecules. However, it becomes expensive in terms of computational cost as in general 85% of the total volume of the solvated system is occupied by the solvent molecules. As most of our RNA complexes were linear in structure, they resulted in solvent system that is too large to perform MDS. Therefore, we used an implicit solvent model as alternative to the explicit solvent simulation. Implicit solvent simulations are computationally less expensive, as the polar and non-polar effects of the solvent molecules are averaged without explicitly including each of them in the calculation. Many other previous studies have successfully applied the implicit solvent model in nucleic acid simulations (63–65).

In the MDS production run, those RNA complexes that were stable after 100 ps were further simulated for another 400 ps. These short duration MD production simulations have been used previously also to determine the stability of nucleic acids (65,66). For the MDS production run, we considered a complex as being stable as long as there is any hydrogen bond present between mRNA and miRNA strands.

The results of the MDS of selected RNA triplexes suggest that the TFE as well as the free energy gain values are important parameters for segregating non-functional candidates of synergistic target regulation. We conclude, that in stable RNA complexes the target mRNA is persistently blocked from translation through the firm hybridization with the pair of cooperative miRNAs.

### Triplex structure and energy profile determine efficiency of cooperative gene repression

To simulate the dynamics of the complex species we used an ODE-based modeling approach because it is suitable for describing the mechanistic details of biochemical reaction systems in terms of temporal dynamics of the involved components.

There exists a small number of ODE-based models that describe gene regulation by individual miRNAs (67–69). In our own previous work we proposed the first mathematical model for cooperative target regulation by pairs of miRNAs (3). In the present study, we refined the model and used predicted equilibrium concentrations and complex-free energies for model parameterization, i.e. for defining complex association and dissociation rates, respectively.

We computed target steady-state concentrations for different synthesis rates (expression profiles) of the cooperating miRNAs. The results of our simulations support the notion that functional miRNA target interactions, in this case the interaction of two cooperating miRNAs and a mutual target mRNA, depend on a low TFE structure.

### Cooperative target regulation—a phenomenon with relevance for cancer?

To identify pathways which tend to be effected by cooperative miRNA regulation we conducted a pathway enrichment analysis using the Database for Annotation, Visualization and Integrated Discovery (DAVID; (70)). For triplexes with experimental support we received predominant enrichment in cancer-specific KEGG pathways (i.e. prostate cancer; small cell lung cancer; bladder cancer, chronic myeloid leukemia, colorectal cancer, glioma, melanoma, pancreatic cancer and endometrial cancer) and some signalling pathways also relevant in cancer as well. This can be explained by the high number of miRNA-target interactions that are validated in the context of cancer related studies. Therefore, we repeated the analysis for the group of triplexes with lowest free energy values (TFE ≤ -41.24kcal/mol) and high predicted triplex equilibrium concentrations (contriplex > 50nM) which we refer to as the set of high-confidence RNA triplexes (this set can be accessed from the Download section in our TriplexRNA database). Still, the results contained many cancer pathways (i.e. prostate cancer, acute myeloid leukemia, renal cell carcinoma, colorectal cancer and melanoma) and some related biological processes (e.g. regulation of actin cytoskeleton, focal adhesion and MAPK signalling), which would suggest some relevance of miRNA-cooperativity in cancer. Data generated in the pathway enrichment analysis (including *P*-values and fold enrichments) are included in the Supplementary Excel file.

## CONCLUSIONS

The main result of our work is the described workflow which can be used to identify RNA triplexes and to determine whether these are functional in terms of cooperative target regulation by two miRNAs. Our analysis demonstrated that beyond the seed site distance there are more triplex features necessary for a functional RNA triplex: first of all a stable local structure (low TFE) with preserved seed bindings; second, a strong binding affinity (high equilibrium probability); third, a strong thermodynamic stability and forth, low triplex dissociation and degradation rates.

Our analysis provides evidence that the phenomenon of cooperative target regulation by miRNA pairs is a common cellular mechanism in animals that facilitates enhanced and fine-tuned target regulation to meet the requirements of given cellular contexts. Our results support the idea that synergistic target repression can lead to a higher specificity in target regulation and that an efficient repression of genes often requires two or more miRNAs (2,69). Single miRNAs typically induce only mild repression to their targets (1). Synergistic target regulation, therefore, provides a molecular means to overcome this restraint.

There may also exist complexes involving more RNA species, e.g. in the case of clustered miRNA-target sites (71). This and other unexplored phenomena show that we are still far from understanding the whole spectrum of mechanisms in post-transcriptional gene regulation.

However, by expanding our knowledge about miRNA cooperation and by validating RNA triplexes relevant in human disease regulation, we may be able to design new therapeutic strategies of RNA interference with higher specificity (72). Nevertheless, our proposed workflow can be applied in other species as well.

## AVAILABILITY

### Database of human RNA triplexes

The data generated in this study were archived in a relational database (MySQL server version 5.5.22) and are freely accessible under the URL: www.sbi.uni-rostock.de/triplexrna. Data access is facilitated by CGI scripts (written in Python v2.7.2). Furthermore, the database is equipped with a RESTful interface for programmatic access, facilitated by the Bottle web framework for Python (http://bottlepy.org/).

### Mathematical model of cooperative target regulation

This model was deposited in BioModels Database (73) and assigned the identifier MODEL1402210000. The MDS movies were submitted to the Database of Simulated Molecular Motions (DSMM; (74)).

## SUPPLEMENTARY DATA

Supplementary Data are available at NAR Online.

SUPPORTING INFORMATION
