# Supplementary material for: Cooperative gene regulation by microRNA pairs and their identification using a computational workflow
Source: Nucleic Acids Res. 2014 May 28;42(12):7539–52. doi: 10.1093/nar/gku465 (PMC4081082; doi:10.1093/nar/gku465)
Supplement: SUPPORTING INFORMATION [file supp_42_12_7539__index.html]

Cooperative gene regulation by microRNA pairs and their identification using a computational workflow — SUPPORTING INFORMATION 

# Cooperative gene regulation by microRNA pairs and their identification using a computational workflow

## SUPPORTING INFORMATION

**Files in this Data Supplement:**

- Supplemental Data
