# Supplementary material for: Cooperative gene regulation by microRNA pairs and their identification using a computational workflow
Source: Nucleic Acids Res. 2014 May 28;42(12):7539–52. doi: 10.1093/nar/gku465 (PMC4081082; doi:10.1093/nar/gku465)
Supplement: SUPPORTING INFORMATION [file supp_gku465_nar-03703-n-2013-File009.zip › Supplementary_material_NAR_rev2.docx]

# Cooperative gene regulation by microRNA pairs and their identification using a computational workflow

Ulf Schmitz^1^, Xin Lai^2^, Felix Winter^1^, Olaf Wolkenhauer^1,3^, Julio Vera^2^, Shailendra K. Gupta ^1,4^

^1^Department of Systems Biology and Bioinformatics, University of Rostock, Rostock, Germany

^2^Laboratory of Systems Tumor Immunology, Department of Dermatology, Faculty of Medicine, University of Erlangen-Nuremberg, Erlangen, Germany

^3^Stellenbosch Institute for Advanced Study (STIAS), Wallenberg Research Centre at Stellenbosch University, Stellenbosch, South Africa

^4^Department of Bioinformatics, CSIR-Indian Institute of Toxicology Research, 226001 Lucknow, India

## miRanda target predictions and the mirSVR score

miRanda was one of the first developed miRNA-target prediction algorithms [1]. It uses a modified Smith-Waterman algorithm (position weighted local alignment algorithm) in that complementarity towards the 5′end of the miRNA is rewarded (scoring factor for the first 11 positions) and position specific empirically defined rules have been applied. Furthermore, miRanda identifies a target site as being conserved if a miRNA independently matches an orthologous site in the same position UTRs in a related species (sequence similarity threshold dependent on the evolutionary distance of the species). Likewise, structure prediction and thermal stability calculation is used for scoring putative miRNA-target pairs.

Recently, a new scoring system which is based on a regression approach for predicting the likelihood of target repression by a miRNA was proposed [2]. The mirSVR score applied to miRanda predictions is based on a support vector regression classifier that was trained with data from miRNA transfection experiments. Sequence, contextual and structural features of predicted miRNA target sites taken from down-regulated target mRNAs have been incorporated into the scoring system which correlates with the extent of down-regulation.

## Structural conformations of 2D RNA triplex models

The predictions revealed four classes of local structural conformations: (i) the canonical triplex (no intra- nor inter-molecular base pairing except for the expected hybridization between the miRNAs and their designated binding sites); (ii) the triplex with target self-complementarity (intra-molecular base pairing in the target sequence); (iii) the triplex with miRNA self-complementarity (intra-molecular base pairing in at least one miRNA sequence); and (iv) the triplex including miRNA-miRNA hybridization (inter-molecular base paring between both involved miRNAs). See Supplementary Figure S4 for an illustration of the different structural conformations. In this context we found that the canonical triplex (Supplementary Figure S4A) and the triplex with target self-complementarity (Supplementary Figure S4B) form typically more stable structures based on their triplex free energy (Supplementary Figure S9, left). Furthermore, we observed that when the canonical triplex structure is formed typically more energy is gained as compared to the other structural conformations (Supplementary Figure S9, right). Of note, we also found that the canonical triplex tends to have higher equilibrium concentrations than other structural conformations (see Box-Whiskers plot in Supplementary Figure S10).

These findings are substantiated by the fact that in the group of triplexes with lower free energy values (TFE ≤ -41.24kcal/mol) and high predicted triplex equilibrium concentrations (contriplex > 50nM) canonical triplexes form the largest fraction ( 68.6%), followed by triplexes with target self-complementarity (18.8%), and triplexes with miRNA self-complementarity (10.5%). The smallest fraction is formed by triplexes that involve miRNA-miRNA hybridization (2.1%). Based on this we conclude that these two conformations are more effective in terms of cooperative miRNA regulation, i.e. stronger target repression will be achieved. Interestingly, when we performed a functional enrichment analysis we found target genes incorporated in these triplexes to be enriched in cancer related pathways (data not shown).

## A kinetic model of miRNA cooperativity

We developed a kinetic model using ODEs to determine target gene repression through the influence of synergistic regulation by pairs of miRNAs. We considered the formation of duplexes by a target mRNA and a miRNA, the formation of a triplex by the target mRNA and two miRNAs and the disassociation of the duplexes and the triplex (Supplementary Figure S6). The model consists of the following equations:

| $\frac{dprotein}{dt}=k_{syn}^{protein}\cdot mRNA-k_{deg}^{protein}\cdot protein$ | [1] |
| --- | --- |
| $\frac{dmRNA}{dt}=k_{syn}^{mRNA}\cdot{TF}_{mRNA}-k_{deg}^{mRNA}.mRNA-k_{ass}^{{duplex}_{1}}\cdot mRNA\cdot{miRNA}_{1} -k_{ass}^{{duplex}_{2}}\cdot mRNA\cdot{miRNA}_{2}-k_{ass}^{triplex}\cdot mRNA\cdot{miRNA}_{1}\cdot{miRNA}_{2}+k_{dis}^{{duplex}_{1}}\cdot{duplex}_{1}+k_{dis}^{{duplex}_{2}}\cdot{duplex}_{2}+k_{dis}^{triplex}\cdot triplex$ | [2] |
| $\frac{d{miRNA}_{1}}{dt}=k_{syn}^{{miRNA}_{1}}\cdot{TF}_{{miRNA}_{1}}-k_{deg}^{{miRNA}_{1}}\cdot{miRNA}_{1}-k_{ass}^{{duplex}_{1}}\cdot mRNA\cdot{miRNA}_{1}-k_{ass}^{triplex}\cdot mRNA\cdot{miRNA}_{1}\cdot{miRNA}_{2}+k_{dis}^{{duplex}_{1}}\cdot{duplex}_{1} +k_{dis}^{triplex}\cdot triplex$ | [3] |
| $\frac{d{miRNA}_{2}}{dt}=k_{syn}^{{miRNA}_{2}}\cdot{TF}_{{miRNA}_{2}}-k_{deg}^{{miRNA}_{2}}\cdot{miRNA}_{2}-k_{ass}^{{duplex}_{2}}\cdot mRNA\cdot{miRNA}_{2}-k_{ass}^{triplex}\cdot mRNA\cdot{miRNA}_{1}\cdot{miRNA}_{2}+k_{dis}^{{duplex}_{2}}\cdot{duplex}_{2}+k_{dis}^{triplex}\cdot triplex$ | [4] |
| $\frac{d{duplex}_{1}}{dt}=k_{ass}^{{complex}_{1}}\cdot mRNA\cdot{miRNA}_{1}-k_{deg}^{{duplex}_{1}}\cdot{duplex}_{1}-k_{dis}^{{duplex}_{1}}\cdot{duplex}_{1}$ | [5] |
| $\frac{d{duplex}_{2}}{dt}=k_{ass}^{{duplex}_{2}}\cdot mRNA\cdot{miRNA}_{2}-k_{deg}^{{duplex}_{2}}\cdot{duplex}_{2}-k_{dis}^{{duplex}_{2}}\cdot{duplex}_{2}$ | [6] |
| $\frac{dtriplex}{dt}=k_{ass}^{triplex}\cdot mRNA\cdot{miRNA}_{1}\cdot{miRNA}_{2}-k_{deg}^{triplex}\cdot triplex-k_{dis}^{triplex}\cdot triplex$ | [7] |

The model includes rate constants for the synthesis ($k_{syn}^{<mRNA, {miRNA}_{i}>}$) of the target mRNA and the miRNAs (respectively, *mRNA and miRNA_i_*) which depend on the presence/activation of the corresponding transcription factors (*TF_mRNA_* and *TF_miRNAi_*), while the synthesis of the target protein ($k_{syn}^{protein}$; *protein*) depends on the presence of free/ transcriptionally active target mRNA. Likewise, the degradation of these molecular species was also considered ($k_{deg}^{<mRNA, miRNA, protein>}$). MiRNAs and the target mRNA are consumed upon duplex and triplex formation ($k_{ass}^{duplex}; k_{ass}^{triplex}$) which results in a reduction of free miRNA/mRNA concentration. Besides, we considered the dissociation of these complexes into their constituent parts as well as their degradation ($k_{dis}^{<duplex, triplex>}; k_{deg}^{<duplex, triplex>}$).

After model construction, we used the predicted complex’s potential energy (CPE) values from step four of the workflow and the equilibrium concentrations of the involved molecules and complexes computed by a partition function algorithm (step five of the workflow), to characterize these model parameter values (see Methods section in the main manuscript and [3] for details).

To our knowledge, no experiments have been performed so far to measure the association and dissociation velocities of the duplexes and triplexes formed by miRNA pairs and target mRNAs. Thus we considered the CPE value as determinant for complex’s thermodynamics stability as suitable for characterizing the disassociation rate constants of the complexes ($k_{dis}^{<duplex, triplex>}$). We also considered the predicted equilibrium concentrations, which reflect probabilities for complex formation, as suitable for characterizing the association rate constants of the complexes ($k_{ass}^{<duplex, triplex>}$). To make these parameters comparable, we normalized them using the highest CPE and equilibrium concentration. By doing so, both the association and dissociation rates constants are constrained between 0 and 1.

For simplicity, the synthesis and degradation rate constants of the molecular species involved were fixed to the value 1, which makes the nominal steady states of the molecular species equal to 1. By doing so, the computed protein expression level clearly indicates if the translation is repressed by miRNAs, in case of a steady state value <1; or not otherwise. The parameter values obtained for each of the selected examples are listed in Supplementary Table S1.

## Supplementary Figures


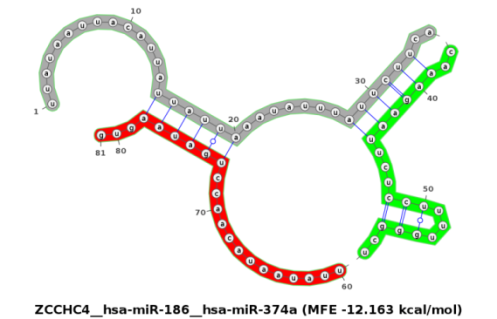


Supplementary Figure S1. Local secondary structure lacking one seed binding.

In the predicted secondary structure of this RNA triplex (ZCCHC4::miR-186::miR-374a) the seed binding is conserved only for miR-186 but not for miR-374a. Therefore, the triplex lacks one important determinant for effective miRNA target regulation. Thus, it is unlikely to observe an enhanced target repression by cooperatively acting miRNAs.


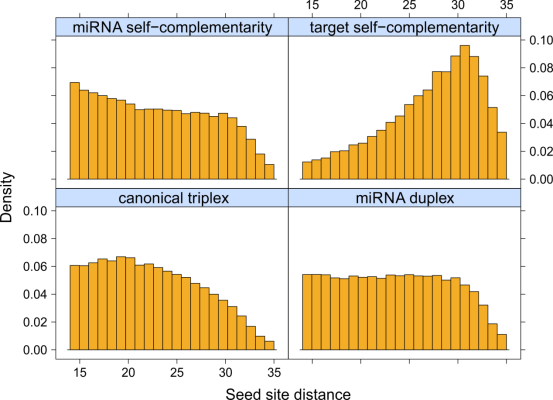


Supplementary Figure S2. Distance frequency distribution.

These histograms show the distribution of the identified distances between the target sites of cooperating miRNAs. Each histogram represents one of the four structural conformations identified in the predicted secondary structures of RNA triplexes. A prerequisite for cooperation according to [4] is a distance of 13-35nt between the target sites. Interestingly, one can observe a clear tendency towards larger seed site distances in the structural pattern target self-complementarity, as these allow the target sequence to self-fold in the region between the two miRNA hybridization sites. In structural pattern, frequencies of seed distances are gradually ascending toward a peak at 31 nt before they start to decline (top right). In contrary, canonical triplexes tend to be more compact and have shorter intermediary target sequences, therefore frequencies of distinct seed site distances start to decline earlier, namely from 21nt onwards (bottom left).


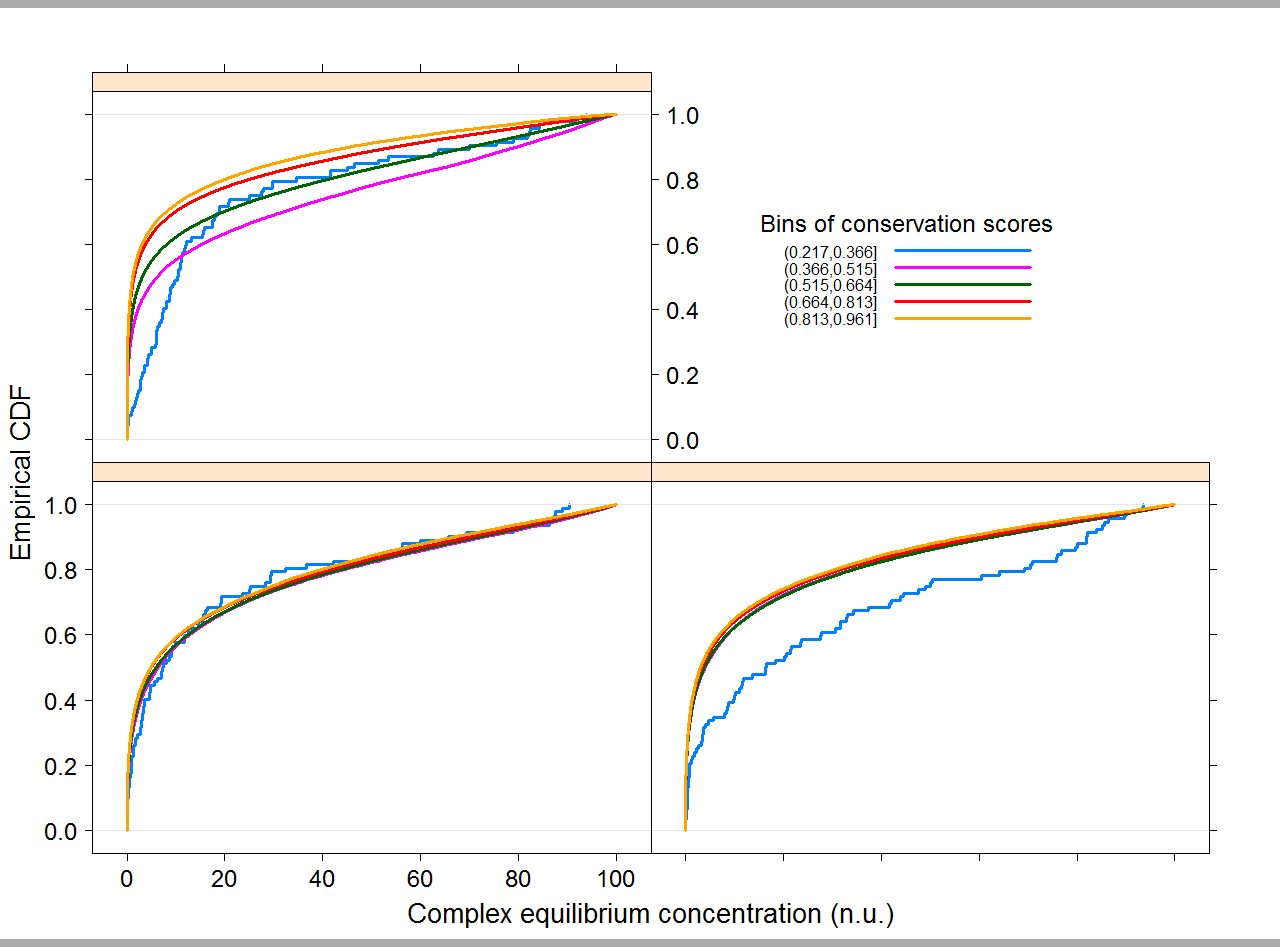


Supplementary Figure S3. Cumulative distribution functions of complex concentrations separated by degree of conservation.

Top: The plot in the upper panel shows the empirical cumulative distribution function (ECDF) of predicted triplex concentrations (association rates) based on the conservation of the inherent miRNA target sites. Conservation scores were split into 5 bins (as defined in the legend). Values for the conservation score are between 0 (no conservation) and 1 (strong conservation) and are based on the scoring system applied in the miRanda algorithm (PhastCons score). Interestingly, triplexes with strongly conserved miRNA target sites tend to have lower equilibrium concentrations (yellow and red lines), while triplexes possessing weakly conserved target sites have higher concentrations by trend (green and pink lines). It might be that miRNAs with strongly conserved target sites are effectively regulating their targets, whereas weakly conserved target sites are often non-functional and therefore depend on the support by a second proximate miRNA target site. Besides, the number of triplexes with very weak conservation is comparably low (n=92) therefore we didn’t consider it in the interpretation of the results. Bottom: In contrary, conservation dependent equilibrium concentrations of mRNA-miRNA duplexes (left and right) are equally distributed.


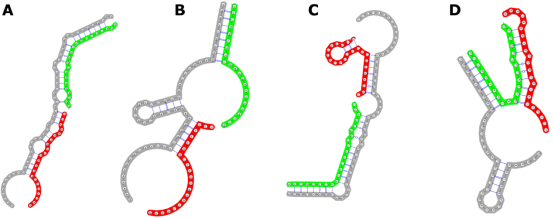


Supplementary Figure S4. Conformations of RNA triplex secondary structures.

**A:** Canonical RNA triplex; **B:** RNA triplex with target self-complementarity; **C:** RNA triplex with miRNA self-complementarity; and **D:** RNA triplex including miRNA-miRNA hybridization. The involved molecules are color coded as follows: target mRNA (grey); first regulatory miRNA (red); and second miRNA (green).


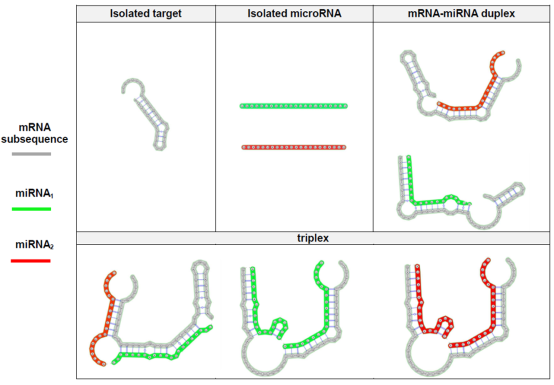


Supplementary Figure S5. Complex species considered in the equilibrium concentration calculations.

The partition function algorithm implemented in the NUPACK software package was used to compute equilibrium concentrations for the monomer and complex species (depicted in this table) that may arise from a given RNA triple.


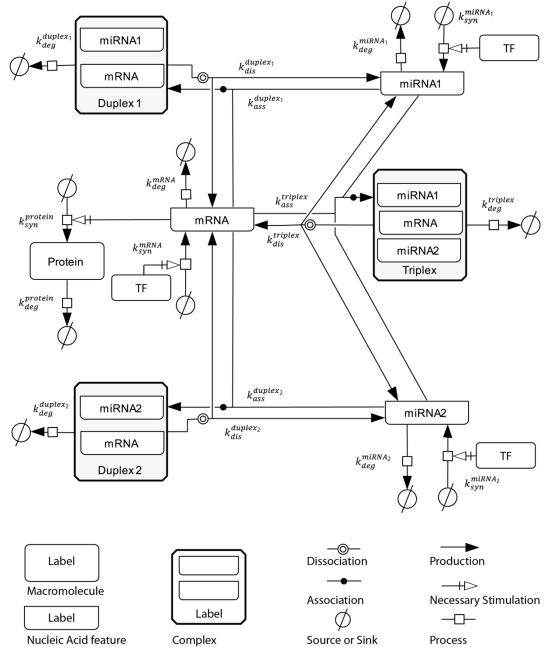


Supplementary Figure S6. Graphic representation of the kinetic model.

The process of target gene repression by a pair of cooperating miRNAs is realized in two manners: (i) the formation of duplexes by the involved miRNAs and the target mRNA; and (ii) the formation of a triplex by the mRNA and the two miRNAs. The figure is presented in systems biology graphical notation (SBGN), which is a standard graphical representation for biochemical networks. Each line is labeled with the name of the corresponding rate constant of the kinetic model. At the bottom we included a legend of symbols used in the diagram.


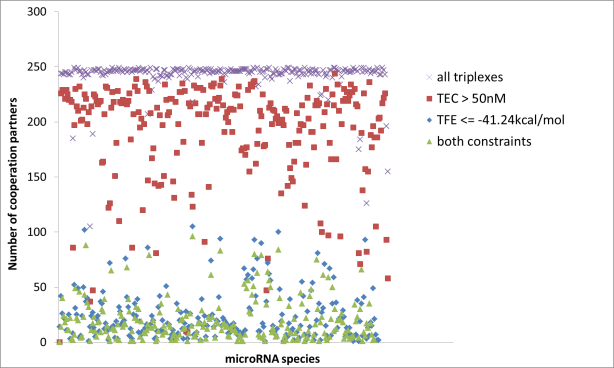


Supplementary Figure S7. Scatterplot showing the number of cooperation partners per miRNA.

In this study we used conserved miRNAs only. In case no further constrains are applied except the target site distance the predictions suggest that miRNAs team up with almost all other miRNAs on some target to conduct cooperative gene repression (purple crosses). The parameters that we propose for the identification of functional RNA triplexes, however, lead to a reduction in putative cooperation partners per miRNA. For example, one can apply a threshold for the triplex equilibrium concentration (e.g., TEC >50nM; red quadrats) or a cut-off for the triplex free energy value (e.g. TFE ≤ -41.24kcal/mol; blue diamonds). Most reliable results are achieved by applying both constraints (green triangles).

Supplementary Figure S8. Workflow for the construction of the 3D structure of mRNA-miRNA complexes

**A:** The secondary structure of RNA triplexes was predicted by the *mfe* tool from the NUPACK software package. The concatenated RNA sequences and the secondary structure in dot-bracket format were submitted to RNAComposer, an automated RNA 3D structure modeling server (http://rnacomposer.ibch.poznan.pl). **B:** Initial 3D structure of the RNA triplex as one large molecule consisting of mRNA (grey), miRNA_1_ (green) and miRNA_2_ (red). **C:** Deletion of phosphodiester bonds between adjoining nucleotides of (i) mRNA and miRNA_1_, and (ii) miRNA_1_ and miRNA_2_; and capping of first nucleotide of mRNA, miRNA_1_ and miRNA_2_. **D:** Assigning CharmM27 force field and geometry optimization to obtain final mRNA + miRNA_1_ + miRNA_2_ triplex.


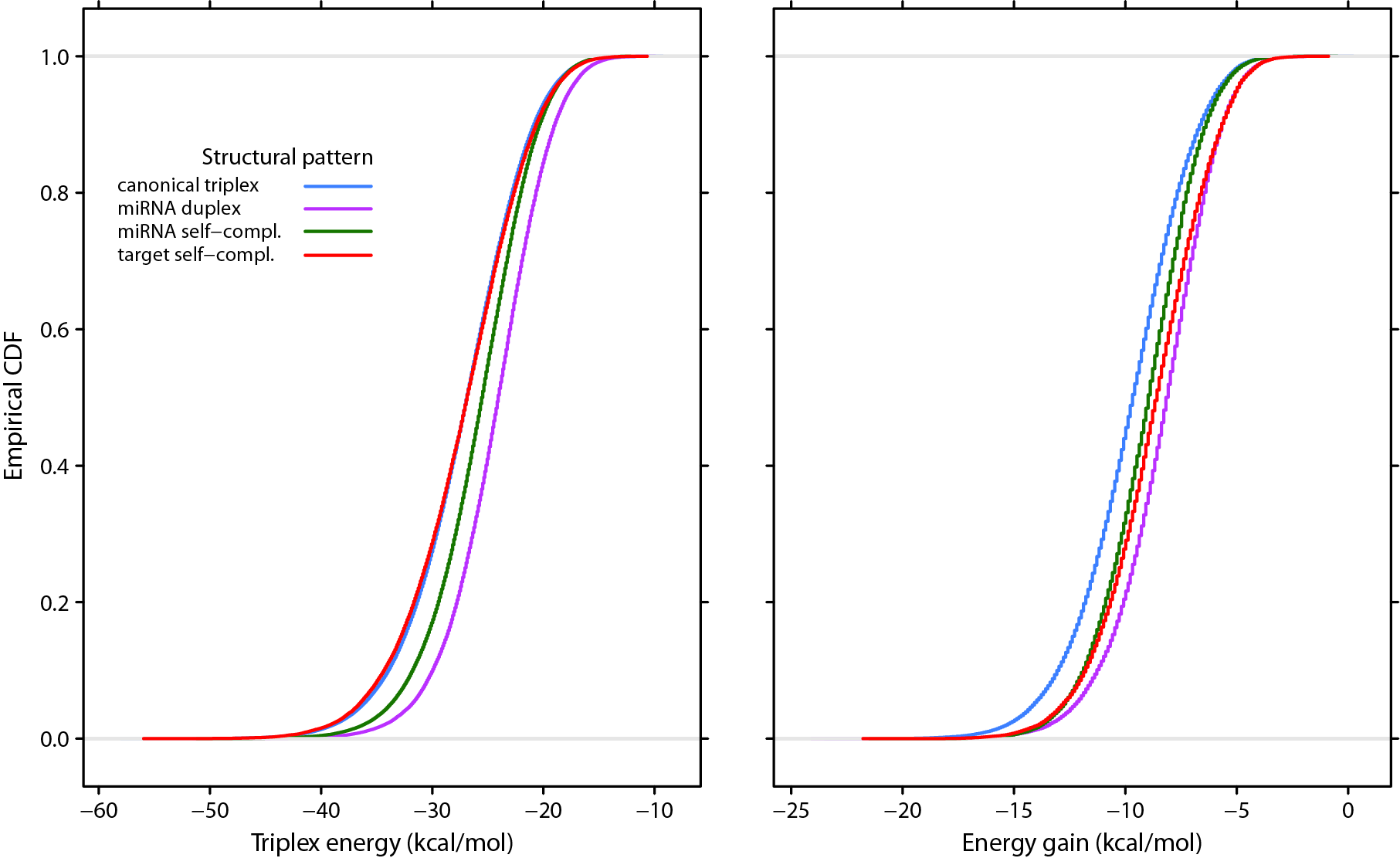


Supplementary Figure S9. Empirical cumulative distribution functions of RNA triplex free energies and free energy gains.

**Left:** There is a clear trend of canonical triplexes (blue line) and triplexes with target self-complementarity (red line) towards lower TFE values and thus thermodynamically more stable complexes. **Right:** The advantage of canonical triplex formation becomes even more evident when the cumulative distribution functions of the free energy gained by triplex formation are compared. Here, solely the canonical triplexes exhibit a benefit in terms of gained energy compared to the other structural conformations.


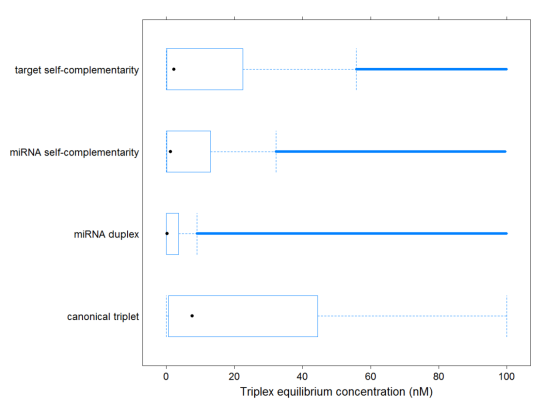


**Supplementary Figure S10. Box-Whiskers plot of predicted triplex equilibrium concentrations.**

The plot demonstrates that RNA triples forming a canonical triplex structure tend to have higher predicted equilibrium concentrations compared to the other structural conformations. Boundaries of the boxes represent 1^st^ and 3^rd^ quartile respectively, while the dot stands for the median of the triplex concentration distribution.


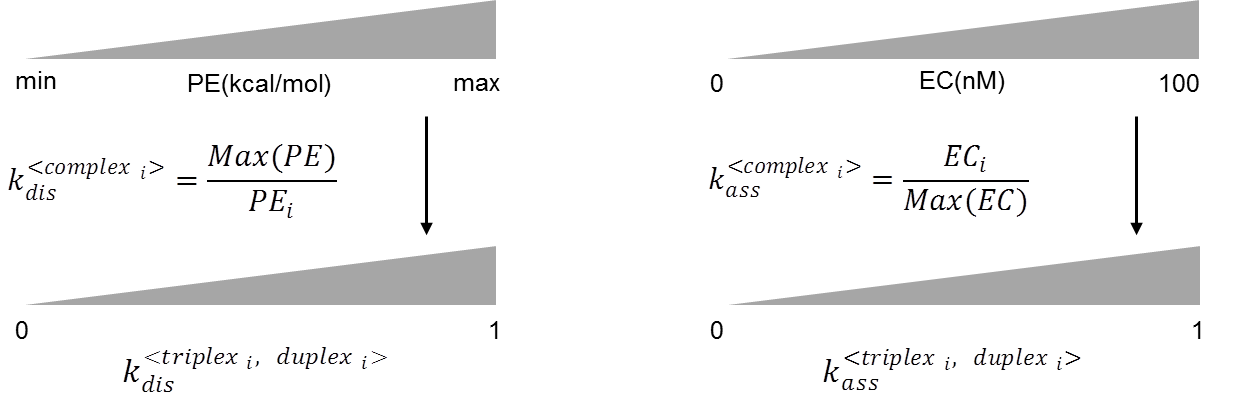


Supplementary Figure S11. Parameter characterization and normalization.

For each of the exemplary cases, we characterized the corresponding parameters using the potential energies (PE) of the complexes calculated in the molecular dynamics simulation and the equilibrium concentrations (EC). Furthermore, the characterized parameter values were normalized using the depicted equations (middle), which ensure that the obtained parameter values for the association and disassociation rate constants of the complexes are constrained in the range [0 1].

## Supplementary tables

Supplementary Table S1. Parameter values of the kinetic model for selected cases.

These parameter values were used for simulating gene repression by cooperative miRNAs as shown in Supplementary Figure S11. All the parameters have normalized units.

|  | $\boldsymbol{k}_{\boldsymbol{ass}}^{\boldsymbol{duplex}_{\boldsymbol{1}}}$ | $\boldsymbol{k}_{\boldsymbol{dis}}^{\boldsymbol{duplex}_{\boldsymbol{1}}}$ | $\boldsymbol{k}_{\boldsymbol{ass}}^{\boldsymbol{duplex}_{\boldsymbol{2}}}$ | $\boldsymbol{k}_{\boldsymbol{ass}}^{\boldsymbol{triplex}}$ | $\boldsymbol{k}_{\boldsymbol{dis}}^{\boldsymbol{duplex}_{\boldsymbol{2}}}$ | $\boldsymbol{k}_{\boldsymbol{dis}}^{\boldsymbol{triplex}}$ |
| --- | --- | --- | --- | --- | --- | --- |
| ***ZNF121*** | 0.0022 | 0.2016 | 0.0022 | 7.2e-06 | 0.2016 | 0.1665 |
| ***HTRA2*** | 0.0070 | 1.0000 | 0.0070 | 2.9e-05 | 1.0000 | 0.3904 |
| ***RPS6KA5*** | 0.0004 | 0.2647 | 0.0007 | 4.3e-06 | 0.2407 | 0.1902 |
| ***ABT1*** | 0.0830 | 0.1088 | 2.2e-06 | 0.9170 | 0.1265 | 0.0949 |
| ***MUC1*** | 0.0000 | 0.1271 | 0.0164 | 0.9836 | 0.1334 | 0.0897 |
| ***EDA2R*** | 0.0005 | 0.1093 | 0.0003 | 0.9991 | 0.1087 | 0.0782 |
| ***C19orf69*** | 0.9505 | 0.5011 | 0.0003 | 0.0001 | 0.5011 | 0.3478 |
| ***KAT2B*** | 0.8225 | 0.3047 | 0.0012 | 0.0001 | 0.2999 | 0.2516 |
| ***NPHP1*** | 0.2112 | 1.0000 | 0.0008 | 2.7e-05 | 0.9146 | 0.6524 |
| ***GOLM1*** | 0.0006 | 0.2342 | 0.0164 | 0.9830 | 0.2715 | 0.1560 |
| ***CCDC3*** | 0.0005 | 0.2500 | 1.3e-05 | 0.9995 | 0.2410 | 0.1878 |
| ***PLXNB1*** | 0.0000 | 0.2314 | 3.4e-07 | 0.9999 | 0.4699 | 0.1814 |

## References

1. Enright AJ, John B, Gaul U, Tuschl T, Sander C, Marks DS: **MicroRNA targets in Drosophila.** *Genome Biol* 2003, **5**:R1.

2. Betel D, Koppal A, Agius P, Sander C, Leslie C: **Comprehensive modeling of microRNA targets predicts functional non-conserved and non-canonical sites.** *Genome Biol* 2010, **11**:R90.

3. Dirks RM, Bois JS, Schaeffer JM, Winfree E, Pierce NA: **Thermodynamic Analysis of Interacting Nucleic Acid Strands**. *SIAM Review* 2007, **49**:65–88.

4. Sætrom P, Heale BSE, Snøve O, Aagaard L, Alluin J, Rossi JJ: **Distance constraints between microRNA target sites dictate efficacy and cooperativity.** *Nucleic Acids Res* 2007, **35**:2333–2342.
